# Molecular docking studies of some selected gallic acid derivatives against five non-structural proteins of novel coronavirus

**DOI:** 10.1186/s43141-021-00120-7

**Published:** 2021-01-25

**Authors:** Haruna Isiyaku Umar, Bushra Siraj, Adeola Ajayi, Tajudeen O. Jimoh, Prosper Obed Chukwuemeka

**Affiliations:** 1 Department of Biochemistry, School of Sciences, Federal University of Technology, Along Owo-Ilesha Express Way, P.M.B. 704, Akure, Ondo State Nigeria; 2 Ioncure Tech Pvt. Ltd., Delhi, 110085 India; 3 Dr. Zafar H. Zaidi Center for Proteomics, University of Karachi, Karachi, Pakistan; 4 Faculty of Pharmaceutical Sciences, Department of Pharmacognosy and Pharmaceutical Botany, Chulalongkorn University, Bangkok, Thailand; 5 Department of Biochemistry, Habib Medical School, Islamic University in Uganda, P. O. Box 7689, Kampala, Uganda; 6 Department of Biotechnology, School of sciences, Federal University of Technology, Akure, Ondo State Nigeria

**Keywords:** In silico, Novel coronavirus, Druglikeness, Gallic acid derivatives, Molecular docking, SARS-COV-2, Non-structural proteins, Molecular interactions, Binding energy

## Abstract

**Background:**

The World Health Organization has recently declared a new coronavirus disease (COVID-19) a pandemic and a global health emergency. The pressure to produce drugs and vaccines against the ongoing pandemic has resulted in the use of some drugs such as azithromycin, chloroquine (sulfate and phosphate), hydroxychloroquine, dexamethasone, favipiravir, remdesivir, ribavirin, ivermectin, and lopinavir/ritonavir. However, reports from some of the clinical trials with these drugs have proved detrimental on some COVID-19 infected patients with side effects more of which cardiomyopathy, cardiotoxicity, nephrotoxicity, macular retinopathy, and hepatotoxicity have been recently reported. Realizing the need for potent and harmless therapeutic compounds to combat COVID-19, we attempted in this study to find promising therapeutic compounds against the imminent threat of this virus. In this current study, 16 derivatives of gallic acid were docked against five selected non-structural proteins of SARS-COV-2 known to be a good target for finding small molecule inhibitors against the virus, namely, nsp3, nsp5, nsp12, nsp13, and nsp14. All the protein crystal structures and 3D structures of the small molecules (16 gallic acid derivatives and 3 control drugs) were retrieved from the Protein database (PDB) and PubChem server respectively. The compounds with lower binding energy than the control drugs were selected and subjected to pharmacokinetics screening using AdmetSAR server.

**Results:**

4-O-(6-galloylglucoside) gave binding energy values of − 8.4, − 6.8, − 8.9, − 9.1, and − 7.5 kcal/mol against Mpro, nsp3, nsp12, nsp13, and nsp15 respectively. Based on the ADMET profile, 4-O-(6-galloylglucoside) was found to be metabolized by the liver and has a very high plasma protein binding.

**Conclusion:**

The result of this study revealed that 4-O-(6-galloylglucoside) could be a promising inhibitor against these SAR-Cov-2 proteins. However, there is still a need for further molecular dynamic simulation, in vivo and in vitro studies to support these findings.

## Background

The World Health Organization has declared the most recent coronavirus (COVID-19) a pandemic and a global emergency that causes a global outbreak since early this year after emerging from Wuhan, Hubei province of China in late December of 2019 [1, 2]. This viral disease is caused by severe acute respiratory syndrome 2 (SARS-COV-2); a spherical-shaped, single-stranded, positively sensed RNA virus with a genome size of approximately 30,000 base pairs consisting of about eleven open reading frames (ORFs) that encodes numerous proteins (structural and non-structural) involved in the viral life cycle [2–4]. The proteins result from the expression of the viral mRNA and sgRNAs subsequent to its entry into the host cell via the priming of the viral spike glycoprotein to angiotensin-converting enzyme 2 (ACE2). The binding of the viral spike glycoprotein to angiotensin-converting enzyme 2 (ACE2) receptor mediates the viral infectivity, hence, serving as the basis for pathogenicity in its host. Once the virus gains entry into the host cell, it liberates its RNA into the cytoplasm subsequently resulting in the translation of its replicase gene. Within the viral replicase gene are two overlapping open reading frames flagged ORF1a/1b. The translation of the ORFs results into polyprotein1a and 1ab. The two polyproteins are subsequently cleaved by two cysteine proteinases called main protease or 3-chymotrypsin-like protease (Mpro/3CLpro) and papain-like protease (PLpro) releasing 16 non-structural proteins (nsp1 to 16). Among these nsps are the papain-like protease (nsp3), main protease (nsp5), RNA-dependent RNA polymerase (RdRp/nsp12), helicase (nsp13), and nsp15 (NendoU) which have been viewed to be viable antiviral drug targets [4, 5].

Papain-like protease (PLpro) is involved in cleavage at the N-terminus of replicase polyproteins to release nsp1, nsp2, and nsp3, also generating replicase complex to allow viral spread [5, 6]. This enzyme is a multifunctional one; aside from polyproteins processing, maturation, and forming replication-transcription complex, it interferes with the host anti-viral immune response by modifying host cellular proteins of immune relevance after their translation [5, 7]. Then, the main protease otherwise called nsp5, cleaved itself from the polyproteins and then cleaved the same polyproteins on 11 distinct sites yielding nsp4 to nsp16 [5, 8]. Interestingly, this process leads to the maturity of the nsps [5, 8]. Also, RNA-dependent RNA polymerase (RdRp/nsp12) is an enzyme of high pertinence that involves in the synthesis of complementary RNA strands with the aid of other nsps such as nsp7 and nsp8 playing a critical role in replication and transcription of the virus [4, 5, 9]. Helicase (nsp13) as well, unwinds the duplex RNA, and also adds a 5′-terminal cap to the viral RNA, thus allowing its recognition for translation and playing a key role in nuclear export, stability, and splicing of the viral mRNA [4, 9]. Finally, Nsp15 (NendoU or EndoU) is an endonuclease that prevents the host immune apparatus to detect the virus via viral RNA cleavage and degradation at the polyuridine sequence region, thereby forming 2′-3′cyclic phosphodiester product [3, 4, 10].

Due to the worldwide outbreak of COVID-19, pharmaceutical companies, government institutions, private organizations, and biotechnological firms are under burdened to produce drugs and vaccines against the ongoing pandemic. As a result, drugs such as azithromycin, chloroquine (sulfate and phosphate), hydroxychloroquine, dexamethasone, favipiravir, remdesivir, interferon-alpha, ribavirin, sofospuvir, ivermectin, and lopinavir/ritonavir have been compassionately used for treatment [11]. Even though significant data are available from clinical randomized trials that could give clear-cut clinical guidance on their use, dosage, or time for treatment or prophylaxis [12], there were several reports on the side effects of these therapies on COVID-19 patients more of which cardiomyopathy, cardiotoxicity, nephrotoxicity, macular retinopathy, and hepatotoxicity [12–14] are the prominent ones. Thus, this has prompted the quest for better and readily available options to thwart this viral malady.

Gallic acid (GA) and its derivatives are one of the abundant plant chemicals with diverse pharmacological aptitudes that can aid in the management of several diseases such as inflammatory, cardiovascular, respiratory, gastrointestinal, and metabolic diseases [15, 16]. Our interest in gallic acid derivatives were based on their pharmacological aptitudes, abundance in nature, and most of all, had not been used for any study to evaluate them against the etiological agent of the recent coronavirus outbreak. Although there are numerous studies on gallic acid and its derivatives against different diseases such as antibacterial, antifungal, and antiviral diseases [17, 18]; nonetheless, no study has reported its use as a therapeutic compound against COVID-19. The derivatives of gallic acid include propyl gallate, lauryl gallate, octyl gallate, theaflavin, tannic acid, (−)-epigallocatechin gallate (EGCG), (−)-epigallocatechin (EGC), (−)-epicatechin gallate (ECG), and (+)-epicatechin (EC) [18, 19]. Realizing the uniqueness of its potentials to treat various diseases, we have considered a subset of its derivatives in this study to understand their mechanistic as inhibitors against the aforementioned viral non-structural proteins via various computational approaches. However, recent studies have revealed the relevance of in silico techniques to find newer and potential therapeutics in drug discovery and development [20–23]. Thus, an alternative novel evidence based on a molecular docking approach could become dexterous to the advancement of novel drug for the management/treatment of this on-going pandemic. In this study, molecular docking was employed to evaluate some selected gallic acid derivatives as antiviral aptitude against SARS-COV-2. We looked into their binding energies (kcal/mol) and molecular interactions with five key important proteins/enzymes of SARS-COV-2, namely, nsp3, nsp5, nsp12, nsp13, and nsp14. Also, we evaluated the pharmaco-activities of the best hit compounds after the docking experiment in silico.

## Methods

### Materials used for the in silico study

The softwares used to execute this present in silico work are Python Prescription 0.8 (PyRx) used for optimizing ligands and for molecular docking since it abhors AutoDock Vina module; UCSF-Chimera© (version 1.13) software used for protein target preparation for docking; PyMol Molecular Graphic system version 2.4.1 used for preparing protein-ligand complexes after docking and for 3D visualization; and Discovery Studio 2016 and LigPlot^+^ were used for 2D visualization of protein-ligand complexes. All softwares were run on a personal computer, HP brand with a processor of Intel (R) Celeron (R) CPU N3060 @ 1.6GHz, 4GB RAM, and 500GB hard disk.

### Selection and optimization of ligands

Gallic acid and fifteen (15) derivatives of gallic acid were selected with three (3) control drugs approved globally to combat COVID-19 (dexamethasone, hydroxychloroquine, and remdesivir). Three-dimensional (3D) structures of these ligands were retrieved from PubChem server (https://pubchem.ncbi.nlm.nih.gov/compound) in simple data format (SDF) and were optimized using Open babel in PyRx (version 0.8) which converted the ligands energetically to the most stable structures using merk molecular force field 94 (MMFF94).

### Drug likeness screening of ligands

The drug likeliness of all the ligands was assessed by the Lipinski’s rule of five parameters (molecular weight < 500 Da, no more than 5 hydrogen bond donors, no. of hydrogen bond acceptors should be less than 10, and AlogP should not be greater than 5). The Lipinski’s rule of five parameters were obtained from the ADMETSar server [24]. Physicochemical properties and toxicity risks assessment prediction was done via the OSIRIS property explorer software (https://www.organic-chemistry.org/prog/peo/). This was done by uploading the respective compounds’ SMILES into the web server and software.

### Protein targets selection and preparation

Main protease (Mpro/nsp5), papain-like protease (Plp/nsp3), RNA-dependent RNA polymerase (RdRp/nsp12), helicase (nsp13), and uridylate-specific endoribonuclease (NendoU/nsp15) of SARS CoV 2 were selected for this study. The 3D crystal structures of the receptors were retrieved from the Protein Data Bank (PDB) (www.pdb.org/pdb) with PDB IDs of 6 LU7 [8], 6W9C [7], 6 M71 [25], 6XEZ [9], and 6VWW [10] respectively. Chain A of the protein receptors was selected and cleaned from heteroatoms, then prepared for docking using the Dock preparation tool of UCSF-Chimera© (version 1.13) software (http://www.cgl.ucsf.edu/chimera). The receptors were minimized base on the AMBER force field in UCSF-Chimera software setting the steepest gradient to 100 and conjugated gradient to 10 [26].

### In silico docking protocol validation

Validation of the docking protocol was carried out to substantiate the exactness and dependability of the docking results. The purpose is to correctly reproduce the binding model and the molecular interaction of the co-crystalized ligand of the experimentally crystalized protein structures in this present in silico studies. Accordingly, the native ligands of all the X-ray proteins were separated from the protein and then prepared for docking via UCSF-Chimera [26]. The ligands were subsequently docked back into the receptors’ active sites using the Auto Dock Vina module of PyRx. The docked complexes were aligned with the X-rayed resolved crystals of proteins bearing the co-crystalized ligand to produce the root mean square deviation (RMSD) value using PyMOL molecular visualizer and also viewing the molecular interactions that results using LigPlot^+^ [27].

### Molecular docking

The molecular docking was executed using a flexible docking protocol as described by Trott and Olson [28] with slight modifications. Accordingly, Python Prescription 0.8, a suite comprising of Auto Dock Vina, was utilized for the molecular docking analysis of the selected ligands with our target proteins. The protein data bank, partial charge, and atom type (PDBQT) files of the proteins were generated (using their previously created PDB files as inputs). All other parameters were kept as default except for the grid box which was adjusted base on the active sites of each protein molecule (see Table 1), and all bonds contained in ligand were allowed to rotate freely, making the receptor rigid [28]. Once the molecular docking experiments were completed and 10 configurations for each protein-ligand complex were generated for all the phytocompounds, text files of scoring results were also produced for the purpose of manual comparative analysis. The lowest binding energy (BE, kcal/mol) and root mean square deviation (RMSD) conformation was considered as the most suitable docking pose. Throughout this in silico investigation, an exhaustiveness of 10 was used for docking, and the number of modes set to 10 so as to achieve more accurate and reliable results. The interaction between ligands and proteins was then prepared, visualized, and analyzed using PyMOL and Discovery Studio 2016 respectively [1, 30].

**Table 1:** Grid box parameters selected and active site of the target proteins

S/N	Target proteins	Center grid box (XYZ), Å	Dimension (XYZ), Å	Active site amino acid residues
1.	Papain-like protease, Nsp3 (PDB ID: 6W9C)	− 39.05 × 39.62 × 32.04	33.50 × 28.55 × 29.87	**Cys111, His272, Asp286,** Gly286, Trp106, Gly271, His73, Arg140, and Asn109 [7]
2.	Main protease, Nsp5 (PDB ID: 6 LU7)	−14.85 × 14.923 × 69.59	25.02 × 27.98 × 30.87	Thr25, Cys44, Thr26, **His41,** Met49, Tyr54, Phe140, Leu141, Gly143, **Cys145**, Asn142, His163, His164, Met165, Ser144, Glu166, Pro168, His172, Val186, Asp187, Arg188, Gln189, Phe185, Thr190, and Gln192 [29]
3.	RNA-Dependent RNA polymerase, Nsp12 (PDB ID: 6 M71)	123.944 × 136.894 × 128.548	20.84 × 18.27 × 25.00	Asp618, Ser759, Asp760, and Asp761 [25]
4.	Helicase, Nsp13 (PDB ID: 6XEZ)	−13.89 × 15.19 × −73.25	29.92 × 25.83 × 24.80	Lys288, Ser289, Asp374, Glu375, Gln404, and Arg567 [9]
5.	Nidoviral Uridylate-specific endoribonuclease, NendoU, Nsps15 (PDB ID: 6VWW)	− 91.15 × 21.86 × −30.63	20.62 × 25.00 × 24.23	**His235, His250, Lys290**, Thr341, Thr343, and Ser294 [10]

### ADMET prediction

ADMET (Adsorption, Distribution, Metabolism, Excretion, and Toxicity) is important to analyze the pharmacodynamics of the proposed molecule which could be used as a drug. ADMETSar and SWISSADME servers were used to predict the ADMET properties of the compounds with the best hits after molecular docking analysis [24, 31, 32]. SMILES of the ligands from PubChem (https://pubchem.ncbi.nlm.nih.gov/compound/) were uploaded onto the search bar of the servers and were predicted.

## Results and discussion

### Druglikeness, physicochemical properties, and toxicity risk assessments

The results of the druglikeness of the ligands selected for this work are presented in Table 2. The molecular weight of the compounds was found to be within the acceptable range except remdesivir with MW 602.59. None of the compounds had a lipophilicity value above 5. Five molecules exceed the number of hydrogen bond acceptors required. In the same vein, six compounds exceeded the acceptable limit for the number of hydrogen bond donors. Finally, two compounds violate the rule while five compounds violated two parameters.

**Table 2:** Druglikeness prediction according to Lipinski’s rule of gallic acid and its derivatives using the admetSAR server

S/N	Compounds	Molecular weight (< 500)	AlogP (< 5)	H-Bond acceptor (< 10)	H-Bond donor (< 5)	Violations
1.	3-O-β-D-Glucopyranoside (3-glucogallic acid)	332.26	− 2.03	9	7	1
2.	3-O-(6-Galloylglucoside)	484.37	−1.04	13	9	2
3.	3-O-Dodecanoyl (3-lauroylgallic acid)	352.43	4.62	5	3	0
4.	3-O-Methyl gallic acid	184.15	0.80	4	3	0
5.	4-O-(6-Galloylglucoside)	484.37	−1.04	13	9	2
6.	4-O-Methyl gallic acid	184.15	0.80	4	3	0
7.	Epicatechin gallate	458.38	2.23	11	8	2
8.	Epicatechin	290.27	1.55	6	5	0
9.	Epigallocatechin gallate	458.38	2.23	11	8	2
10.	Epigallocatechin	306.27	1.25	7	6	1
11.	Gallic acid	170.12	0.50	4	4	0
12.	Lauryl gallate	338.44	4.88	5	3	0
13.	Octyl gallate	282.34	3.32	5	3	0
14.	Propyl gallate	212.20	1.37	5	3	0
15.	Pyrogallol	126.11	0.80	3	3	0
16.	Resorcinol	110.11	1.10	2	2	0
	**Control drugs**					
17.	Dexamethasone	392.47	1.90	5	3	0
18.	Hydroxychloroquine	335.88	3.78	4	2	0
19.	Remdesivir	602.59	2.31	13	4	2

In addition, the physicochemical and toxicity risk assessments were carried out using OSIRIS property explorer. The results are presented in Table 3 with parameters such as solubility, topological surface area (TPSA), druglikeness, drug score, mutagenicity, tumorigenicity, reproductive effect, and irritation. All compounds showed good solubility between −0.63 and − 4.99. Eight compounds were predicted to have poor druglikeness properties; whereas four showed very low drug scores (below 0.20). The toxicity risk assessment shows that gallic acid, propyl gallate, pyrogallol, resorcinol, and remdesivir pose a toxicity risk.

**Table 3:** Physicochemical properties and toxicity risks assessment employing the OSIRIS Property Explorer (https://www.organic-chemistry.org/prog/peo/)

S. no	Compounds	Solubility (Log S)	TPSA (Å)	Druglikeness	Drug score	Mutagenic	Tumorigenic	Reproductive effect	Irritants
1	3-O-β-D-Glucopyranoside (3-glucogallic acid)	−0.63	177.1	−3.44	0.48	No	No	No	No
2	3-O-(6-Galloylglucoside)	−1.32	243.9	1.77	0.71	No	No	No	No
3	3-O-Dodecanoyl (3-lauroylgallic acid)	− 4.04	104.0	−25.23	0.3	No	No	No	No
4	3-O-Methyl gallic acid	−1.06	86.99	1.1	0.85	No	No	No	No
5	4-O-(6-Galloylglucoside)	−1.32	243.9	1.99	0.72	No	No	No	No
6	4-O-Methyl gallic acid	−1.05	86.99	1.24	0.69	No	Medium risk	No	No
7	Epicatechin gallate	−2.16	197.3	1.58	0.7	No	No	No	No
8	Epicatechin	−1.76	110.3	1.92	0.87	No	No	No	No
9	Epigallocatechin gallate	−2.16	197.3	1.58	0.7	No	No	No	No
10	Epigallocatechin	−1.47	130.60	1.10	0.82	No	No	No	No
11	Gallic acid	−0.74	97.99	0.12	0.27	**Yes**	No	**Yes**	No
12	Lauryl gallate	−3.87	86.99	−20.89	0.28	No	No	No	No
13	Octyl gallate	−2.79	86.99	−20.89	0.25	No	No	No	**Yes**
14	Propyl gallate	−1.44	86.99	0.54	0.17	**Yes**	**Yes**	**Yes**	No
15	Pyrogallol	−0.73	60.69	−3.5	0.07	**Yes**	**Yes**	**Yes**	**Yes**
16	Resorcinol	−1.02	40.46	− 1.94	0.12	**Yes**	**Yes**	No	**Yes**
	**Control drugs**								
17	Dexamethasone	−3.25	94.83	3.18	0.8	No	No	No	No
18	Hydroxychloroquine	−3.55	48.39	6.54	0.48	**Yes**	No	No	No
19	Remdesivir	−4.99	213.30	−30.39	0.05	No	**Yes**	**Yes**	**Yes**

Solubility, ranging 0 (highly soluble) to −6 (poorly soluble); TPSA, topological polar surface area ≤ 130 Å^2^; druglikeness, scores with positive value are likely to be an oral drug; drug score, 0-1.0

### Molecular docking analysis

Prior to docking, our docking protocol was validated for all target receptors as explained in the “Method” section. This crucial step was carried out in other to provide guarantees of the molecular docking protocols and softwares to accurately deliver correct binding signatures with quality molecular interactions between target receptors and the compounds investigated in this in silico study. The outcome of this exercise is depicted in Figs. 2a, 3, 4, 5, and 6a. This exercise was adjudged to be successful since the docked complexes reproduced the original poses as the native ligands precisely (Figs. 2a, 3, 4, 5, and 6a) with RMSD values of 0.000 Å, 0.533 Å, 0.605 Å, 2.298 Å, and 0.208 Å for Mpro, PLpro, RdRp, helicase, and NendoU respectively. In this present docking work, sixteen ligands (thirteen gallic acid derivatives and three control drugs) were docked against five protein targets in SARS-Cov-2, namely, Mpro, PLp, RdRp, helicase/nsp13, and NendoU Autodock Vina module in-built in Python Prescription suit according to Trott and Olson [28]. The binding energies (BE) that results after the docking experiment are provided in Table 4.

**Table 4:** Binding energies of Ligands docked against Mpro, Plp, RdRp, helicase, and NendoU of SARS-Cov-2

S. No.	Ligands	Binding affinity (kcal/mol)				
		NSP5/MPro	NSP3/PLp	NSP12/RdRp	NSP13/Helicase	NSP15/NendoU
1.	4-O-(6-Galloylglucoside)	−7.8	−**6.8**	−**8.9**	−**9.1**	−**7.5**
2.	Epigallocatechin-gallate	−7.7	−5.9	−8.5	−8.5	−6.9
3.	3-O-(6-Galloylglucoside)	−**8.4**	−**6.6**	− 8.4	− 9.0	− 6.8
4.	Epicatechin-gallate	− **8.4**	− 6.1	− 8.3	− 8.4	− 7.2
5.	3-Glucogallic-acid	− 6.7	− 5.6	− 7.1	− 7.9	− 7.0
6.	Epicatechin	− 7.1	−6.0	− 7.1	− 7.6	− 6.8
7.	Epigallocatechin	− 6.9	− 5.7	− 7.1	-7.3	− 6.7
8.	3-O-Methyl-gallic-acid	−5.4	−4.8	− 6.2	− 6.1	− 5.2
9.	Gallic-acid	− 5.6	−4.6	− 5.9	− 5.9	−5.0
10.	3-O-Dodecanoyl(3-lauroylgallic-acid)	−5.3	−4.5	−5.8	− 6.4	− 5.7
11.	Lauryl-gallate	− 5.4	−4.1	− 5.5	− 6.1	−5.5
12.	Octyl gallate	−5.8	−5.0	− 5.1	− 6.6	−5.7
13.	4-o-Methylgallic-acid	−5.1	−4.6	−5.0	− 6.0	− 5.1
**Control drugs**						
14.	Dexamethasone	−6.8	−6.4	−**7.5**	− **8.0**	− **7.1**
15.	Remdesivir	−**7.9**	−**6.5**	− 7.3	− 7.4	− 6.7
16.	Hydroxychloroquine	− 6.3	−5.0	−6.1	− 6.8	− 5.8

After the docking was completed, the least binding energy (BE, kcal/mol) and root mean square deviation (RMSD) conformation was considered as the most suitable docking pose of the compounds as presented in Table 4. 3-O-(6-Galloylglucoside) (3O6G) and epicatechin gallate (ECG) produced the least BE of −8.4 kcal/mol when docked with Mpro. Similarly, 3-O-(6-galloylglucoside) and its isomer, 4-O-(6-galloylglucoside) (4O6G) yielded the least BE of −6.8 and −6.6 kcal/mol respectively with PLp. Also, 4-O-(6-galloylglucoside) show the least BE of −8.9, −9.1, and −7.5 kcal/mol when docked with RdRp, nsp13, and NendoU respectively.

### Molecular interaction analysis

**Fig. 1:**
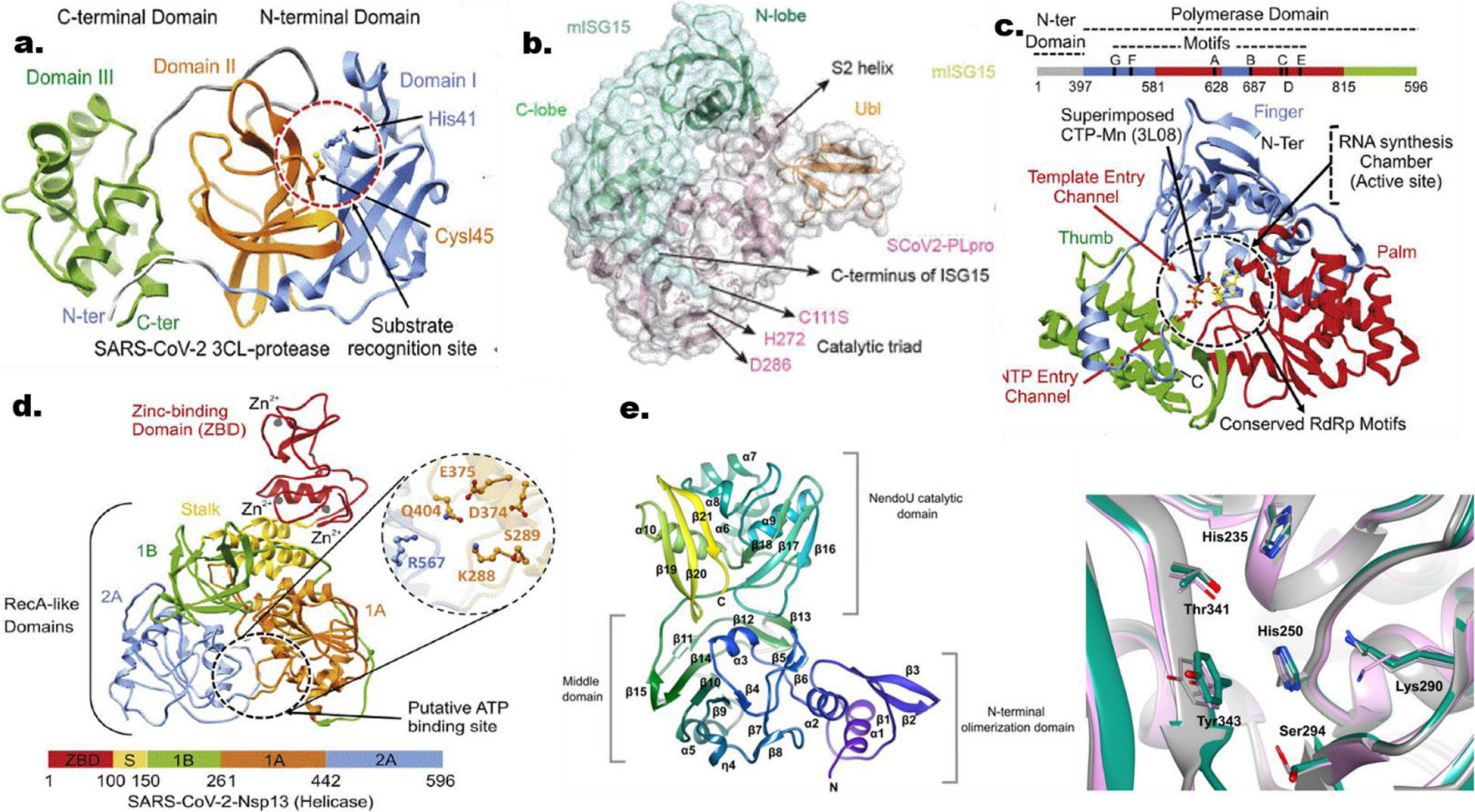
Structures of SARS COV 2 proteins with their respective binding site. (**a**) Main protease (3CL-protease), (**b**) papain-like protease, (**c**) RdRp, (**d**) helicase and NendoU. Sourced from Mirza and Froeyen (2020) Structural elucidation of SARS-CoV-2 vital proteins. 10.1016/j.jpha.2020.04.008 and Kim et al (2020)

**Fig. 2:**
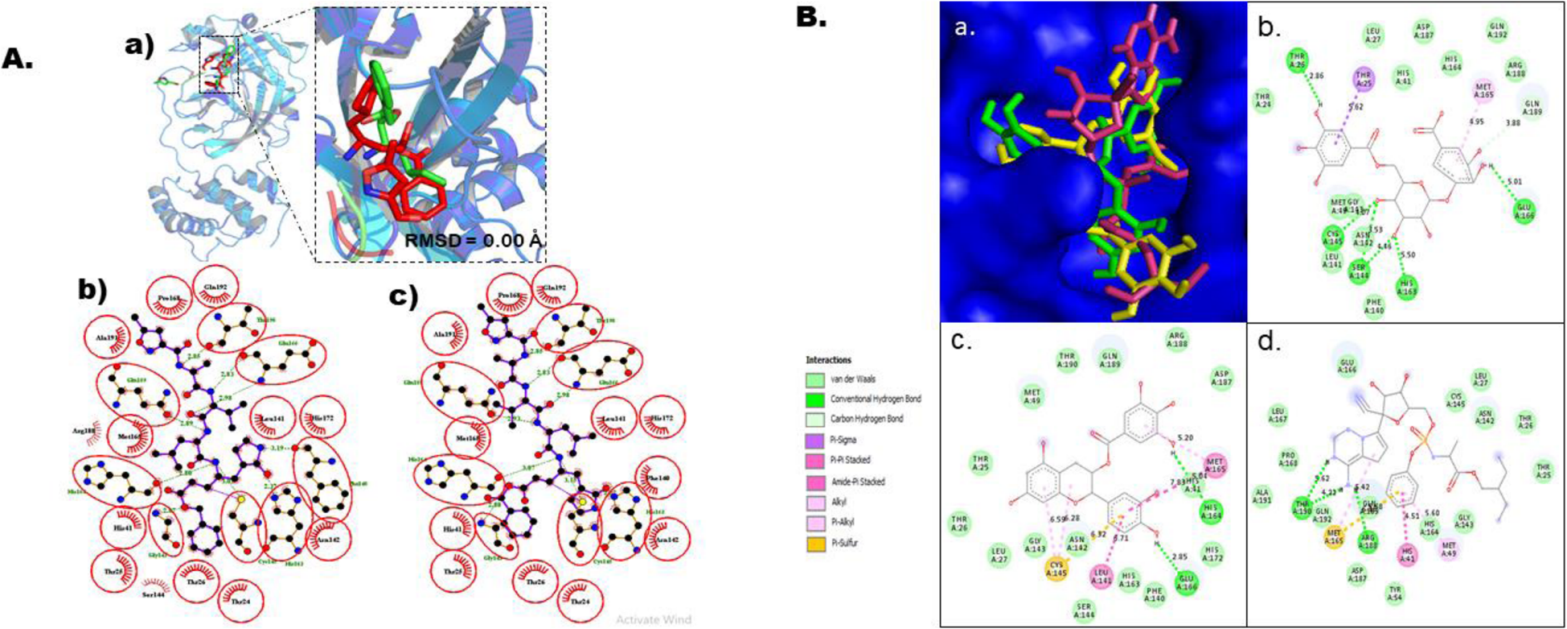
Molecular docking studies of gallic acid derivatives against main protease (Mpro) of SARS-Cov-2. **a** Protocol validation of molecular docking experiment using AutoDock Vina, PyMOL, and LigPlot^+^. (**a**) Comparison of binding modes for re-docked ligand (red) vs. co-crystallized ligand (green) shown as stick representation. Amino acid residues interaction with (**b**) co-crystalized and (**c**) re-docked ligand accomplished in LigPlot^+^. **b** Binding mode and molecular interaction of hit ligands with Mpro. (**a**) Surface representation of Mpro (PDB: 6 LU7) show the binding mode of docked 3-O-(6-galloylglucoside) (yellow), epicatechin gallate (green), and remdesivir (pink). 2D interaction of (**b**) 3-O-(6-galloylglucoside), (**c**) epicatechin gallate, and (**d**) remdesivir

In this current in silico study, gallic acid derivatives were docked against Mpro using Auto Dock Vina. The Mpro used for this investigation was retrieved in its 3D crystal structure (6 LU7), co-crystallized with an inhibitor N-leucinamide, resolved at 2.16 Å, sequence length of 306, and a homodimeric protein [29]. The binding site of Mpro is surrounded by 25 amino acids such as Thr25, Cys44, Thr26, His41, Met49, Tyr54, Phe140, Leu141, Gly143, Cys145, Asn142, His163, His164, Met165, Ser144, Glu166, Pro168, His172, Val186, Asp187, Arg188, Gln189, Phe185, Thr190, and Gln192. Also, the binding site shows a *CysHis* (Cys145 and His41) catalytic dyad [29, 33] (Fig. 1). We found that 3-O-(6-galloylglucoside) and epicatechin gallate had the utmost bound energy of −8.4 kcal/mol (Table 4). This prompted the initiative to visualized their binding pose and bring to fore their interaction patterns with the target protein in relative to the best hit control drug (remdesivir) using PyMol and Discovery Studio visualizer. The compounds were observed to fit into the same cavity of the binding site (Fig. 2b). More also, 3-O-(6-galloylglucoside) was able to produce interaction with five amino acids within the binding pouch of Mpro such as Thr26, Ser144, Cys145, His163, and Glu166 at bond length of 2.86 Å, 4.46 Å, 4.07 Å, 5.50 Å, and 5.01 Å respectively. A pi-sigma bond of length 5.62 Å was also observed between the aromatic side chain of Thr25 and the galloyl’s aromatic ring of 3-O-(6-galloylglucoside), while a pi-alkyl interaction with bond length of 4.95 Å was established between the aromatic side chain of Met165 and the aromatic ring attached to the glucoside. A carbon-hydrogen bond was formed with Gln189. The remaining interaction was by van der Waals with Thr24, Leu27, His41, Met49, Phe140, Leu141, Asn142, Gly143, His164, Asp187, Arg188, and Gln192. However, Glu166 and His164 interacted with epicatechin gallate (ECG) via hydrogen bond at a distance of 2.85 Å and 5.04 Å respectively. Pi-pi and amide-pi stacked interactions emerged between Met165 and Leu141 with ECG at bond lengths of 7.83 Å and 6.71 Å respectively. Thr25, Leu27, Met49, His41, Phe140, His163, Ser144, Gly143, His172, Asp187, Arg188, Gln189, and Thr190 interact with ECG through van der Waals forces. More so, Cys145 interacts with ECG by forming alkyl, pi-alkyl, and pi-sulfur bonds with distance of 6.28 Å, 6.59 Å, and 6.32 Å respectively. Among the control drugs in this study, remdesivir showed a good bound energy of −7.9 kcal/mol. It was able to form three hydrogen bonds with Thr190 and Arg188 at a distance of 5.62 Å, 4.22 Å, and 6.26 Å respectively. Meanwhile, Met165 established pi-alkyl and pi-sulfur bonds with distance of 5.42 Å and 5.68 Å respectively. Met49 and His41 associated with control drug via pi-alkyl and pi-pi stacked bonds with distance of 5.60 Å and 4.51 Å respectively.

**Fig. 3:**
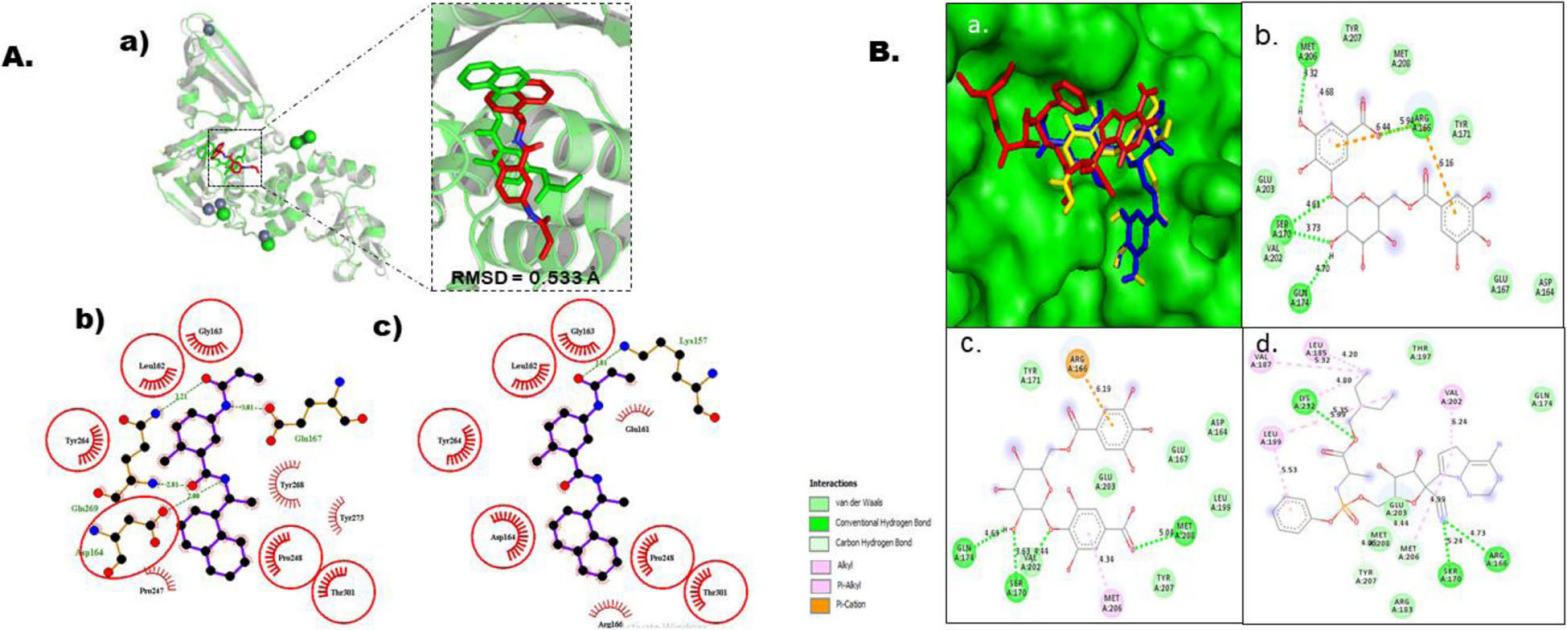
Molecular docking studies of gallic acid derivatives against papain-like protease (PLpro) of SARS-Cov-2. **a** Protocol validation of molecular docking experiment using AutoDock Vina, PyMOL, and LigPlot^+^. (**a**) Comparison of binding modes for re-docked ligand (green) vs. co-crystalized ligand (red) shown as stick representation. Amino acid residues interaction with (**b**) co-crystalized and (**c**) re-docked ligand accomplished in LigPlot^+^. **b** Binding mode and molecular interaction of hit ligands with PLp. (**a**) surface representation of PLp (PDB: 6W9C) show the binding mode of docked 3-O-(6-galloylglucoside) (yellow), 4-O-(6-galloylglucoside) (blue), and remdesivir (red). 2D interaction of (**b**) 3-O-(6-galloylglucoside), (**c**) 4-O-(6-galloylglucoside) and (**d**) remdesivir

The PLpro used for this investigation was retrieved in its 3D crystal structure (6W9C), resolved at 2.70 Å, with sequence length of 317, and a homotrimeric protein [7]. The substrate binding site in PLpro is made up of a canonical cysteine protease catalytic triad (Cys111, His272, and Asp286) (Fig. 1) found at the interface between the thumb and palm domains; other amino acids in the binding site are Trp106, Gly266, Gly271, His73, Arg140, and Asn109 [7]. The in silico docking of PLp with gallic acid derivatives revealed that 3-O-(6-galloylglucoside), 4-O-(6-galloylglucoside), and remdesivir had the utmost binding affinity scores of −6.6, −6.8, and −6.5 kcal/mol respectively (Table 4). Furthermore, the molecular interactions of these inhibitors were predictably ascertained indicating that these inhibitors bind to the same cleft of the catalytic site of our target protein (Fig. 3b). 3-O-(6-Galloylglucoside) with Met206, Arg166, Gln174, and Ser170 formed hydrogen bonds at distance of 3.32 Å, 5.94 Å, 4.70 Å, 3.73 Å, and 4.61 Å respectively. Although Met206 had another interaction with 3O6G via pi-alky bond with a distance of 4.68 Å while two pi-sulfur bonds were observed between Arg166 and 3O6G, Glu203, Val202, Tyr207, Met208, and Tyr171 interacted via van der Waals forces. 4O6G had Gln174, Ser170 and Met208 engaged via hydrogen bonding at distance of 4.69 Å, 3.63 Å, 4.44 Å, and 5.04 Å respectively. Moreover, Arg166 and Met206 were engaged via pi-sulfur and pi-alkyl interactions with 4O6G at distance 6.19 Å and 4.34 Å respectively. Van der Waals force was observed between 4O6G with Va202, Tyr171, Asp164, Glu167, Glu203 and Tyr207. Remdesivir binding was found to involve three H-bonds with Lys232, Ser170, and Arg166 with distance of 5.35 Å, 5.24 Å, and 4.73 Å respectively. Met206 and Val202 established pi-alky bonds with remdesivir at distance of 4.99 Å and 6.24 Å respectively. Lys232, Leu199, Val187, and Leu185 form links with our ligand via an alky-type bond. While Leu199 established a pi-alkyl bond with remdesivir.

**Fig. 4:**
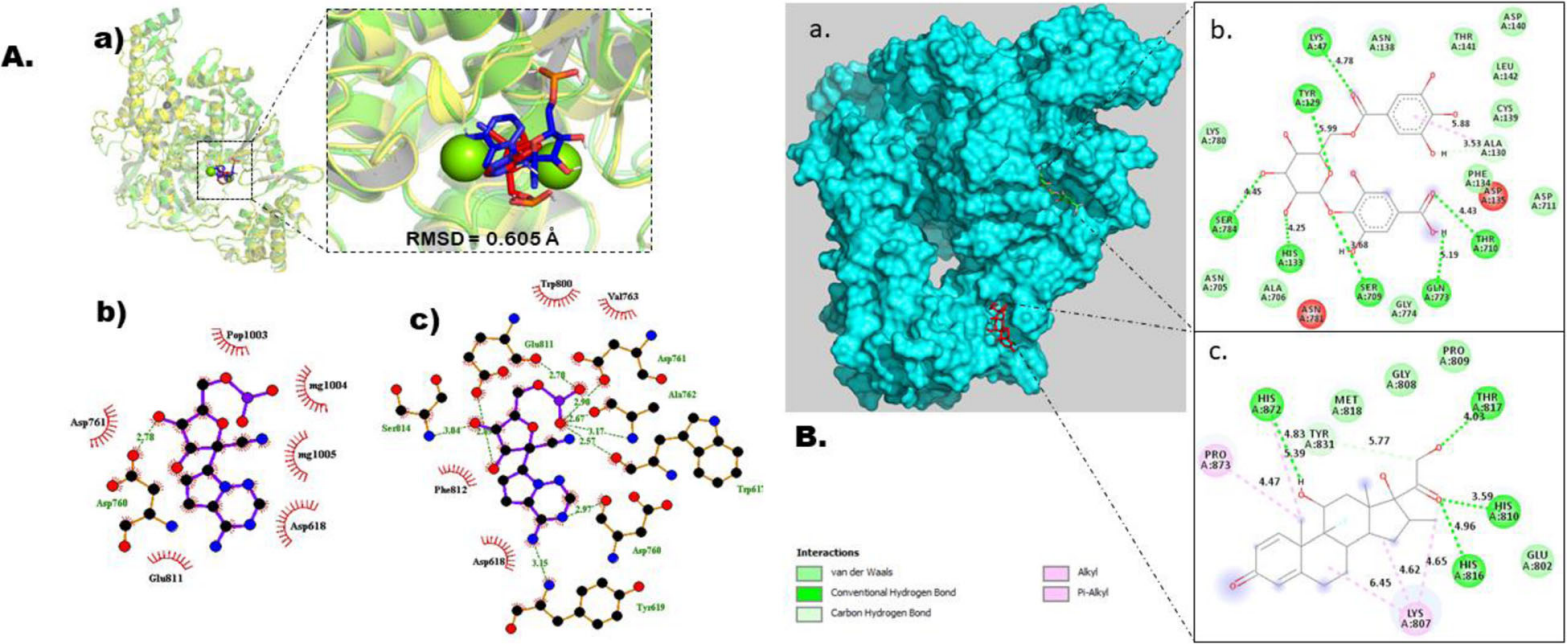
Molecular docking studies of gallic acid derivatives against RNA-dependent RNA polymerase (RdRp) of SARS-Cov-2. **a** Protocol validation of molecular docking experiment using AutoDock Vina, PyMOL, and LigPlot^+^. (**a**) Comparison of binding modes for re-docked ligand (blue) vs. co-crystalized ligand (red) shown as stick representation. Amino acid residues interaction with (**b**) co-crystalized and (**c**) re-docked ligand accomplished in LigPlot^+^. **b** Binding mode and molecular interaction of hit ligands with RdRp. (**a**) surface representation of RdRp (PDB: 6 M71) show the binding mode of docked 4-O-(6-galloylglucoside) (green), and dexamethasone (red). 2D interaction of (**b**) 4-O-(6-galloylglucoside) and (**c**) dexamethasone

3D crystal structure of nsp12 was retrieved from protein data bank with PDB code of 6 M71, resolution of 2.9 Å, fused together with nsp8 and nsp7 as cofactors, chain A of the complex and sequence length of 942 amino acids [25]. The active site of nsp12 is conserved in the polymerase motifs A-G (Fig. 1), which comprises of Asp618, Ser759, Asp760, and Asp761 [25]. Total of seventeen gallic acid derivatives was screened for identifying the potential drug against RdRp using molecular docking. All the compounds were evaluated on the basis of their maximum scoring function, and the top ranked compound was selected and analyzed for the interactions with RdRp protein. 4-O-(6-Galloylglucoside) possessed the highest binding affinity of −8.9 kcal/mol showing strong bonding with RdRp (Table 4). The docked conformation of 4-O-(6-galloylglucoside) with their interacting residues is shown in Fig. 4. This compound contributes seven hydrogen bonding interactions with viral RdRp which were observed between Lys47, Tyr129, His133, Ser709, Thr710, Gln773, and Ser784 at distance of 4.78 Å, 5.99 Å, 4.25 Å, 3.68 Å, 4.43 Å, and 5.19 Å respectively. Pi-alkyl interaction with Ala130 at a distance of 5.88 Å. Lys780, Asn705, Ala706, Gly774, Asp711, Phe134, Cys139, Leu142, Asp140, Thr141, and Asn138 interact with 4O6G via van der Waals forces. Dexamethasone exhibits highest −7.5 kcal/mol binding affinity although remdesivir was a known RdRp inhibitor. Within the binding cavity of RdRp, it forms hydrogen bond with His810, Thr817, Tyr816, and His872 at distance of 3.59 Å, 4.03 Å, 4.96 Å, and 5.39 Å respectively. Carbon-hydrogen bond was observed with Tyr831 at a distance of 5.77 Å. Moreover, His872, Pro873, and Lys807 formed alkyl-type interactions at distance of 5.39 Å, 4.47 Å, 4.65 Å, 6.45 Å, and 4.62 Å respectively with control drug. The van der Waals interaction occurred between the drug and Met818, Gly808, Pro809 in addition to Glu802.

**Fig. 5:**
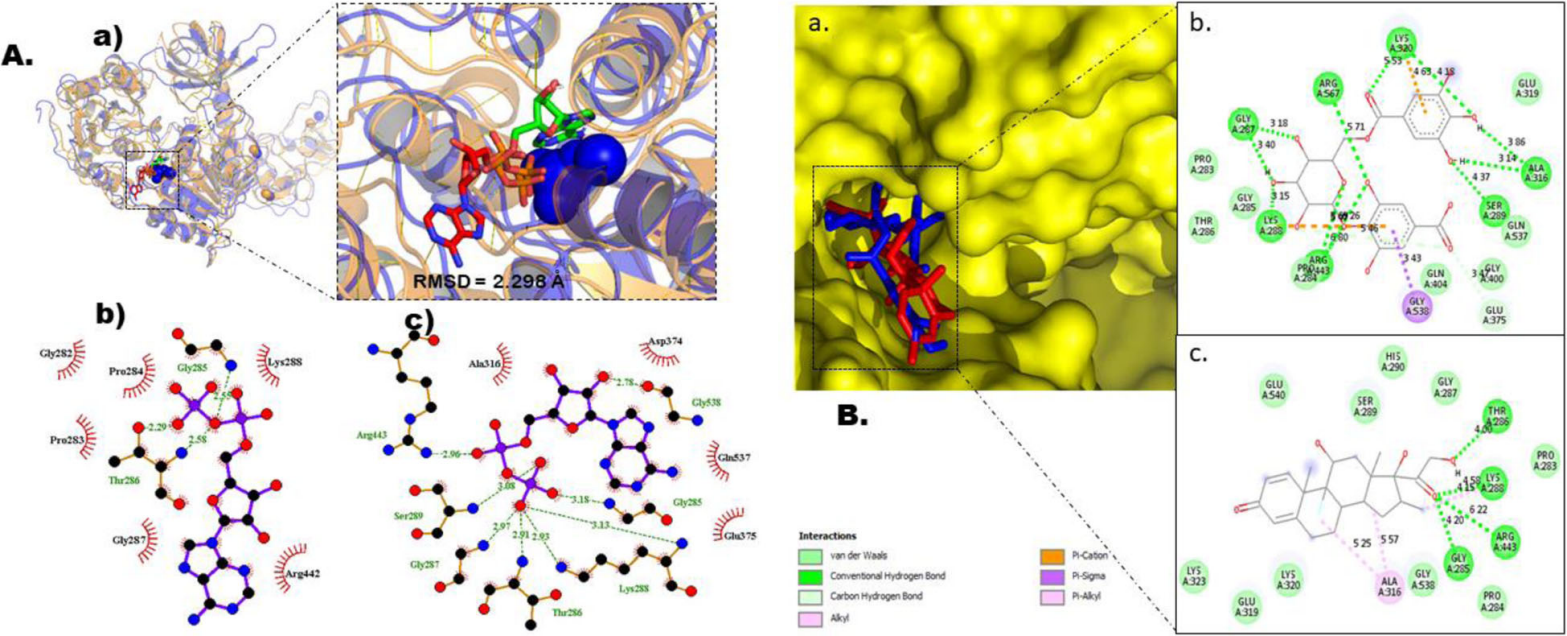
Molecular docking studies of gallic acid derivatives against helicase/Nsp13 (RdRp) of SARS-Cov-2. **a** Protocol validation of molecular docking experiment using AutoDock Vina, PyMOL, and LigPlot^+^. (**a**) Comparison of binding modes for re-docked ligand (green) vs. co-crystalized ligand (red) shown as stick representation. Amino acid residues interaction with (**b**) co-crystalized and (**c**) re-docked ligand accomplished in LigPlot^+^. **b** Binding mode and molecular interaction of hit ligands with Helicase. (**a**) surface representation of Helicase (PDB: 6XEZ) show the binding mode of docked 4-O-(6-galloylglucoside) (blue), and dexamethasone (red). 2D interaction of (**b**) 4-O-(6-galloylglucoside) and (**c**) dexamethasone

The 3D crystal structure was together with RdRp, nsp8, and nsp7 (6XEZ), with resolution of 3.5 Å, sequence length of 605 amino acids and homodimeric [9]. The amino acids of relevance in the binding cavity of nsp13 are Lys288, Ser289, Asp374, Glu375, Gln404, and Arg567 [9] (Fig. 1). Gallic acid derivatives were screened as potential inhibitors of helicase using molecular docking. At the end of the docking study, 4O6G had the best binding affinity of −9.1 kcal/mol. Also, dexamethasone, one of the control drugs in this study fared better than other control drugs with binding affinity of −8.0 kcal/mol (Table 4). 4O6G and dexamethasone occupied similar area in the active portion of helicase (Fig. 5b). The molecular interaction in 2D show the binding signatures maintained by both molecules (Fig. 5b). 4O6G was able to create hydrogen bond with seven amino acids such as Gly287, Lys288, Arg443, Arg567, Lys320, Ala316, and Ser289 at distance of 3.18 Å, 3.40 Å, 3.15 Å, 6.80 Å, 5.69 Å, 6.26 Å, 5.71 Å, 5.53 Å, 4.18 Å, 3.86 Å, 3.14 Å, and 4.37 Å respectively. Also, pi-cation bond was formed with Lys288 and Lys320 at distance of 5.46 Å and 4.63 Å respectively. However, Gly538 interacted with 4O6G via a pi-sigma bond. In addition, van der Waals force form the link between 4O6G and eight amino acids which are Pro283, Gly285, Thr286, Pro284, Gln404, Gly400, Gln537, and Glu319 then Glu375 established a carbon-hydrogen bond with 4O6G at a distance of 3.47 Å. Dexamethasone, interacted with Thr286, Lys288, Arg443, and Gly285 at distance of 4.00 Å, 4.58 Å, 6.22 Å, and 4.20 Å respectively via hydrogen bond. Although, alkyl interaction occurred with Lys288 and Ala316 at distance of 4.15 and 5.57 respectively but Ala316 formed another interaction via pi-alky bond at a distance of 5.25 Å. However, ten amino acids interacted with dexamethasone by van der Waals force. They include Glu540, Ser289, His290, Gly287, Lys323, Glu219, Gly538, Lys320, Pro284, and Pro283.

**Fig. 6:**
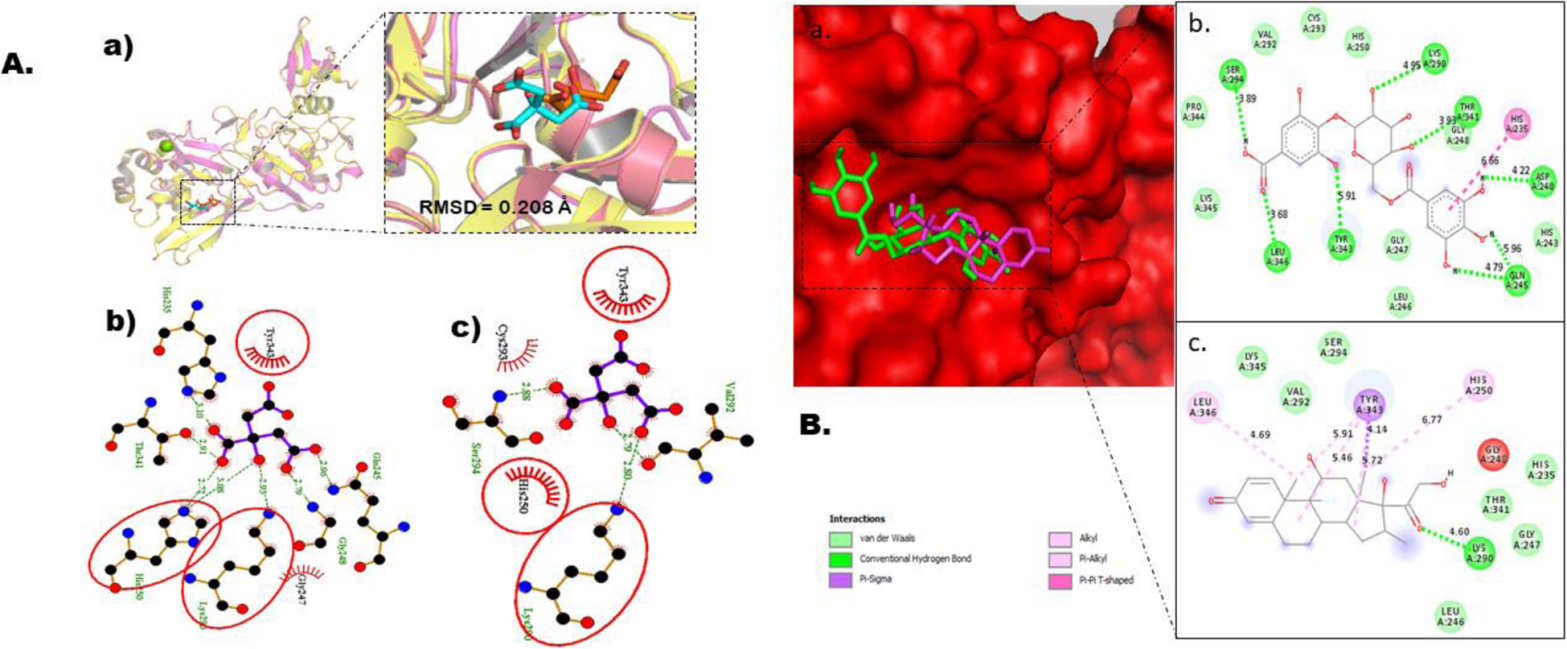
Molecular docking studies of gallic acid derivatives against Nidoviral RNA uridylate-specific endoribonuclease, NSP15 (NendoU) of SARS-Cov-2. **a** Protocol validation of Molecular Docking experiment using AutoDock Vina, PyMOL, and LigPlot^+^. (**a**) Comparison of binding modes for re-docked ligand (red) vs. co-crystalized ligand (cyan) shown as stick representation. Amino acid residues interaction with (**b**) co-crystalized and (**c**) re-docked ligand accomplished in LigPlot^+^. **b** Binding mode and molecular interaction of hit ligands with NendoU. (**a**) Surface representation of NendoU (PDB: 6VWW) show the binding mode of docked 4-O-(6-galloylglucoside) (green), and dexamethasone (pink). 2D interaction of (**b**) 4-O-(6-galloylglucoside) and (**c**) dexamethasone

A 2.20 Å X-ray resolved protein was used in this current work with sequence length of 342 amino acids and hexameric structure [10]. The active cavity of NendoU is made up of His235, His250, Lys290, Thr341, Thr343, and Ser294 [3, 10] (Fig. 1). Therefore, it is of pertinence to evaluate the selected gallic acid derivatives against NendoU using molecular docking technique. 4O6G (−7.5 kcal/mol) and dexamethasone (−7.1 kcal/mol) were found to have the utmost binding affinity among the gallic acid derivatives and the control drugs respectively. Both compounds occupied the same site on the catalytic region of the target protein (Fig. 6b). The binding patterns of both compounds are shown in Fig. 6b-c. 4O6G was able to interact with seven amino acids through hydrogen bond. The amino acids involved are Ser296, Leu346, Tyr343, Lys290, Thr341, Asp240, and Gln245 with distance of 3.89 Å, 3.68 Å, 5.91 Å, 4.95 Å, 3.93 Å, 4.22 Å, 4.79 Å, and 5.96 Å respectively. His235 forms a pi-pi T-shaped bond with 4O6G while Pro344, Lys345, Leu246, Gly247, His243, Gly248, Cys293, His250, and Val292 all interacted with the compound via van der Waals force. However, dexamethasone formed hydrogen bond with Ly290 at a distance of 4.60; Pi-sigma and pi-alkyl bonds with Tyr343 at a distance of 4.14 Å and 5.46 Å respectively; alkyl bond with Leu346, His250, and Tyr343 at distance of 4.69 Å, 6.77 Å, 5.91 Å, and 5.72 Å respectively. Lys345, Ser294, Val292, Gly248, His235, Thr341, Gly247, and Leu246 interacted with the drug via van der Waals force.

### ADMET prediction

3-O-(6-Galloylglucoside), 4-O-(6-galloylglucoside), and epicatechin gallate were screened for their absorption, distribution, metabolism, and toxicity (ADMET) through a web tool called AdmetSAR. Also, dexamethasone and remdesivir were also screened. This in silico toxicity screening of our hit compounds cannot be over emphasized as it will ultimately predict the pharmacokinetics and pharmacodynamics of these compounds in relation to the selected control drugs. As we know, the control drugs are part of the treatment regimen in the fight against this global malady, but there are safety concerns. The results are depicted in Table 4.

In the absorption class, caco-2 permeability, human intestinal absorption, human oral availability, p-glycoprotein inhibition and substrates were predicted. Out of the five molecules, only dexamethasone was predicted to permeant caco-2, epicatechin gallate, dexamethasone, and remdesivir were predicted to be absorbed by the human intestine. None of them were predicted to be orally available but dexamethasone and remdesivir might serve as substrate to p-glycoprotein. Although remdesivir was predicted to inhibit p-glycoprotein. The distribution of these molecules were predicted via two properties (PPB and BBB). All the compounds indicate that they might possibly reach their target site in high to moderate dose as their ability to bind to plasma proteins was predicted to be high. Dexamethasone and remdesivir might be able to reach the central nervous system (CNS) as they were predicted to permeant the blood-brain-barrier (BBB). All the compounds were predicted to be localized in the mitochondria except remdesivir which was predicted to be localized in the lysosomes. The metabolism of these molecules by the human liver was predicted to ascertain whether they can cause harm or not. More also, none of these molecules were predicted to inhibit cytochrome P450 1A2 and 3A4. 3-O-(6-galloylglucoside) and 4-O-(6-galloylglucoside) were predicted to be non-substrate to Cytochrome P450 3A4. None of the compounds were predicted to be substrates or inhibitors of Cytochrome P450 2C9, 2C19, and 2D6. All the compounds were catalyzed by UGT except epicatechin gallate and remdesivir as predicted by the web server. The toxicity was evaluated based on acute oral toxicity, AMES mutagenesis, carcinogenicity, and human hepatotoxicity. None of the compound was predicted to be AMES mutagenic and carcinogens; 4-O-(6-galloylglucoside) and dexamethasone were predicted to be non-toxic to the liver cells. The acute oral toxicity (kg/mol) were also predicted (Table 5).

**Table 5:** ADMET properties of best hits compounds with remdesivir. ADMET was predicted using the AdmetSAR server

Class	Properties	3-O-(galloylglucoside)	4-O-(galloylglucoside)	Epicatechin gallate	Remdesivir	Dexamethasone
**Absorption**	Caco-2 permeability	Negative	Negative	Negative	Negative	Positive
	Human intestinal absorption	Negative	Negative	Positive	Positive	Positive
	Human oral bioavailability	Negative	Negative	Negative	Negative	Negative
	Pgp-inhibitor	Negative	Negative	Negative	**Positive**	Negative
	Pgp-substrate	Negative	Negative	Negative	Positive	Positive
**Distribution**	PPB (plasma protein binding)	Moderately high (95.31%)	Moderately high (94.27%)	High (102.20%)	Very high (118.2%)	High (100%)
	BBB (Blood–brain barrier)	Negative	Negative	Negative	Positive	Positive
**Metabolism**	CYP450 1A2 inhibition	Negative	Negative	Negative	Negative	Negative
	CYP450 3A4 inhibition	Negative	Negative	Negative	Negative	Negative
	CYP450 3A4 substrate	Negative	Negative	Positive	Positive	Positive
	CYP450 2C9 inhibition	Negative	Negative	Negative	Negative	Negative
	CYP450 2C9 substrate	Negative	Negative	Negative	Negative	Negative
	CYP450 2C19 inhibition	Negative	Negative	Negative	Negative	Negative
	CYP450 2D6 inhibition	Negative	Negative	Negative	Negative	Negative
	CYP450 2D6 substrate	Negative	Negative	Negative	Negative	Negative
	CYP inhibitory promiscuity	Negative	Negative	Negative	Negative	Negative
	UGT catalyzed	Positive	Positive	Negative	Negative	Positive
	Sub-cellular localization	Mitochondria	Mitochondria	Mitochondria	Lysosomes	Mitochondria
**Toxicity**	Acute oral toxicity (kg/mol)	2.5726	2.5958	2.3672	3.4279	2.486
	Ames mutagenicity	Negative	Negative	Negative	Negative	Negative
	Carcinogens	Negative	Negative	Negative	Negative	Negative
	Human hepatotoxicity	Positive	Negative	Positive	Positive	Negative

## Discussion

Gallic acid (GA) and its derivatives are one of many phytocompounds with diverse pharmacological propensities that can assist in the management of several diseases such as inflammatory, cardiovascular, respiratory, gastrointestinal, and metabolic diseases caused by bacteria, fungi, and viruses. It is pertinent to note in this present in silico study, compounds were selected based on their pharmacological roles, large quantity in nature, and most significantly, had not been used for any study to evaluate them against the etiological agent of the recent coronavirus outbreak be it in silico, in vitro, nor in vivo. However, there are numerous studies on gallic acid and its derivatives against different diseases such as antibacterial, antifungal, and antiviral diseases [17, 18]; nonetheless, no study have reported its use as a therapeutic compound against the on-going pandemic. Base on resent studies [20–23], computational approaches have been deployed to discover novel and potential small molecules in drug discovery and development.

The compounds and control drugs were screened for their oral druggability via Lipinski’s rule; a rule of thumb that states that for a molecule to be orally active as drug, the molecular weight must not exceed 500 Da, lipophilicity must be below 5, number of hydrogen bond acceptors and donors must not exceed 10 and 5 respectively. The consequence of a molecule exceeding these landmarks might affect its adsorption, metabolism, excretion, and toxicity in the body, making it a poor drug. The physicochemical and toxicity assessment was carried out in silico to fish out some compounds that were perceived to be toxicant; this step buttressed some of the findings of the druglikeness screening using Lipinski’s rule.

The significance of our selected SARS-CoV-2 proteins had earlier been discussed. Main protease (Mpro) is a vital enzyme in SARS COV 2 that acts on polyproteins to give rise to nsp4 to nsp16. Thus, without this action, viral replication and transcription might not take place. The binding site of Mpro is surrounded by 25 amino acids such as Thr25, Cys44, Thr26, His41, Met49, Tyr54, Phe140, Leu141, Gly143, Cys145, Asn142, His163, His164, Met165, Ser144, Glu166, Pro168, His172, Val186, Asp187, Arg188, Gln189, Phe185, Thr190, and Gln192. Also, the binding site shows a *CysHis* (Cys145 and His41) catalytic dyad [29, 33]. The BE of 3O6G and ECG noticed with Mpro might be as a result of the interactions that were established with the two key amino acid residues that formed the catalytic triad in its substrate binding domain (Fig. 2 and Table 4). Another vital protein in viral life cycle with an additional function of innate immune antagonism by cleaving ubiquitin and ISG15 from interferon-α (IFN-α) is the papain-like protease (PLpro) [6]. The substrate binding domain is made up of a canonical cysteine protease catalytic triad (Cys111, His272, and Asp286) (Fig. 1) found at the interface between the thumb and palm domains; other amino acids in these domain are Trp106, Gly266, Gly271, His73, Arg140, and Asn109 [7]. It is noteworthy that none of the hit compounds had interactions with the catalytic triad (Cys111, His272, and Asp286) and might be the reason for the characteristics BE values exhibited by them (Fig. 3 and Table 4).

RNA-dependent RNA polymerase (RdRp/nsp12) is one of the crucial molecular target for developing promising leads against coronavirus since is the central piece of the replication-transcription machinery [9, 25, 34]. The active site of nsp12 is conserved in the polymerase motifs A-G (Fig. 1), comprising of Asp618, Ser759, Asp760, and Asp761 [25]. The BE exhibited by 4O6G and dexamethasone might be ascribed to the interactions that results with key residues that are pertinent to activity of the enzyme. However, helicase, nsp13 as a member of the functional complex with RdRp called replication-transcription complex (RTC) is also crucial in the life cycle of SARS COV 2 [9]. The amino acids of relevance in the binding cavity are Lys288, Ser289, Asp374, Glu375, Gln404, and Arg567 [9] (Fig. 1). The characteristics BE portrayed by 4O6G and dexamethasone might be attributed to their abilities to interact with three key amino acid residues that contributes to the catalytic activity of helicase (Fig. 5 and Table 4). NendoU (nsp15) is key to SARS COV 2 since it is known to interfere with the host immune system by cleaving the double and single stranded RNA at the uridylate region releasing 2′-3′cyclic phosphate end [10]. The active cleft of NendoU is made up of His235, His250, Lys290, Thr341, Thr343, and Ser294 [3, 10] (Fig. 1). The BE exhibited after docking by 4O6G and dexamethasone against NendoU might be linked to the interactions formed with key amino acid residues that are significant to the catalytic activity of the enzyme (Fig. 6 and Table 4).

The interactions reported in this present in silico study, viz., hydrogen bond, hydrophobic interaction (alkyl and pi-typed bonds), and electrostatic interactions (pi-cation and anion), had been reported [27, 35, 36] earlier to provide stability to the protein–ligand complexes and also influence the binding energy values of the hit compounds in complex with all the target proteins. Thus, the available facts from the pool of investigated active compounds related to gallic acid, suggest that 3O6G, 4O6G, and ECG may possess the most in silico inhibitory effect against SARS-CoV-2 through a molecular docking technique. However, these outcomes need to be validated using molecular dynamic simulation, in vitro and in vivo studies. The studies from in silico toxicity profiling (Table 5) revealed that 4-O-(6-galloylglucoside) is better than the rest of the hit compounds but it will require chemosimilar or pharmacophoric modeling that will improve some key parameters relating to absorption and distribution which might be linked to the earlier screening for druglikeness using the Lipinski’s rule of five where it violated the parameters: hydrogen bond donor and acceptor. This is also the same for the remaining two hit compounds. Thus, they could be repurposed as a defense line against this viral malady.

## Conclusion

At present, the greatest threat to global human health is the recent coronavirus outbreak caused by the novel SARS-CoV-2 coronavirus. We performed a molecular docking assay with some selected gallic acid derivatives against five key proteins of SARS-CoV-2 that are found to be vital throughout the viral life cycle and proliferation. These proteins are Nsp3, Nsp5, Nsp12, Nsp13, and Nsp15. From our present in silico study, we observed that three of the selected compounds, namely, 3-O-(6-galloylglucoside), 4-O-(6-galloylglucoside), and epicatechin gallate could be promising inhibitors of the selected SARS-CoV-2 non-structural proteins, and a possible treatment option against the current COVID-19 pandemic. The result, from our present in silico study, also revealed that 4-O-(6-galloylglucoside) had the best binding energies (BE) against Nsp3, Nsp12, Nsp13, and Nsp15 with BE of −6.8, −8.9, −9.1, and −7.5 kcal/mol respectively. However, 3-O-(6-galloylglucoside) and epicatechin gallate show better BE against Nsp5 of −8.4 kcal/mol each. Although the studies from in silico toxicity profiling revealed that 4-O-(6-galloylglucoside) is better than the rest of the hit compounds, thus, could be repurposed as a defense line against this viral malady. However, since this study had been accomplished through molecular docking methods, there would be need for molecular dynamics simulation of at least 100 ns to validate the outcome of our present in silico study. More so, an actual experiment of the identified compounds in this study for both in-vitro and in-vivo studies with appropriate models is also recommended to further authenticate the findings from this study.

## Data Availability

We declare that all the data generated are included in this study.

## Abbreviations

ADMET: Adsorption, distribution, metabolism, excretion and toxicity
BE: Binding energy
COVID-19: Coronavirus disease 19
Mpro: Main protease
NSP: Non-structural proteins
ORF: Open reading frame
PLpro: Papain-like protease
RdRp: RNA-dependent RNA polymerase
RNA: Ribonucleic acid
SARS-CoV 2: Severe acute respiratory syndrome coronavirus 2

## References

[1] Kamaz Z, Al-jassani MJ, Umar H (2020). Screening of common herbal medicines as promising direct inhibitors of Sars-Cov-2 in silico. Annu Res Rev Biol.

[2] Anjorin AA (2020) The coronavirus disease 2019 (COVID-19) pandemic: a review and an update on cases in Africa. Acian Pacific J Trop Med 13:1–6. 10.4103/1995-7645.281612

[3] Alazmi M, Motwalli O (2020) In silico virtual screening, characterization, docking and molecular dynamics studies of crucial SARS-CoV-2 proteins. J Biomol Struct Dyn 1–11. 10.1080/07391102.2020.1803965 10.1080/07391102.2020.1803965 PMC7484580 32762537

[4] Yoshimoto FK (2020) The proteins of severe acute respiratory syndrome coronavirus - 2 (SARS CoV - 2 or n - COV19), the cause of COVID - 19. Protein J 39(3):198–216. Available from: 10.1007/s10930-020-09901-4 10.1007/s10930-020-09901-4 PMC7245191 32447571

[5] Muhammed Y (2020) Molecular targets for COVID-19 drug development: enlightening Nigerians about the pandemic and future treatment. Biosaf Heal:1–7 https://doi.org/10.1016/j.bsheal.2020.07.002 10.1016/j.bsheal.2020.07.002 PMC7343650 32838282

[6] Shin D, Mukherjee R, Grewe D, Bojkova D, Baek K, Bhattacharya A, Schulz L, Widera M, Mehdipour AR, Tascher G et al (2020) Papain-like protease regulates SARS-CoV-2 viral spread and innate immunity. Nature.:1–31 10.1038/s41586-020-2601-5 10.1038/s41586-020-2601-5 PMC7116779 32726803

[7] Osipiuk J, Azizi S-A, Dvorkin S, Endres M, Jedrzejczak R, JK A, Kathayat RS, Lisnyak VG, Maki SL, Kang S (2020). Structure of papain-like protease from SARS-CoV-2 and its complexes with non-covalent inhibitors. bioRxiv.

[8] Jin Z, Du X, Xu Y, Deng Y, Liu M, Zhang B, Li X, Zhang L, Peng C, Duan Y (2020). Structure of M pro from COVID-19 virus and discovery of its inhibitors. Nature.

[9] Chen J, Malone B, Llewellyn E, Grasso M, Shelton PMM, Olinares PDB, Maruthi K, Eng ET, Vatandaslar H, Chait BT (2020). Structural basis for helicase-polymerase coupling in the SARS-CoV-2 replication-transcription complex. Cell.

[10] Kim Y, Jedrzejczak R, Maltseva NI, Wilamowski M, Endres M, Godzik A, Michalska K, Joachimiak A (2020) Crystal structure of Nsp15 endoribonuclease NendoU from SARS-CoV-2. Protein Sci:1–10 10.1002/pro.3873 PMC7264519 32304108

[11] McKee DL, Sternberg A, Stange U, Laufer S, Naujokat C (2020) Candidate drugs against SARS-CoV-2 and COVID-19. Pharmacol Res 157:1–9. https://doi.org/10.1016/j.phrs.2020.104859 10.1016/j.phrs.2020.104859 PMC7189851 32360480

[12] Khan Z, Karatas Y, Rahman H (2020). Anti COVID-19 drugs: need for more clinical evidence and global action. Adv Ther.

[13] Javorac D, Grahovac L, Manić L, Stojilković N, Anđelković M, Bulat Z, Ćosić DĐ, Curcic M, Djordjevic AB (2020) An overview of safety assessment of the medicines currently used in the treatment of COVID-19 disease. Food Chem Toxicol:1–32 10.1016/j.fct.2020.111639 10.1016/j.fct.2020.111639 PMC7372271 32707160

[14] Aggarwal G, Henry BM, Aggarwal S, Bangalore S (2020). Cardiovascular safety of potential drugs for the treatment of coronavirus disease 2019. Am J Cardiol.

[15] Kahkeshani N, Farzaei F, Fotouhi M, Alavi SS, Bahramsoltani R, Naseri R, Momtaz S, Abbasabadi Z, Rahimi R, Farzaei MH (2019). Pharmacological effects of gallic acid in health and disease: a mechanistic review. Iran J Basic Med Sci.

[16] Badhani B, Sharma N, Kakkar R (2015). Gallic acid: a versatile antioxidant with promising therapeutic and industrial applications. RSC Adv.

[17] AL Zahrani NA, El-Shishtawy RM, Asiri AM (2020). Recent developments of gallic acid derivatives and their hybrids in medicinal chemistry: a review. Eur J Med Chem.

[18] Choubey S, Varughese LR a, Kumar V, Beniwal V (2015). Medicinal importance of gallic acid and its ester derivatives: a patent review. Pharm Pat Anal.

[19] Singh PM, Gupta A, Sisodia SS (2018). Gallic acid: pharmacogical promising lead molecule: a review. Int J Pharmacogn Phytochem Res.

[20] Lokhande KB, Ballav S, Yadav RS, Swamy V, Basu S (2020) Probing intermolecular interactions and binding stability of kaempferol, quercetin and resveratrol derivatives with PPAR- γ: docking , molecular dynamics and MM/GBSA approach to reveal potent PPAR-γ agonist against cancer. J Biomol Struct Dyn 1–11. 10.1080/07391102.2020.1820380 10.1080/07391102.2020.1820380 32954977

[21] Lokhande KB, Ballav S, Thosar N, Swamy KV, Basu S (2020) Exploring conformational changes of PPAR-Ɣ complexed with novel kaempferol, quercetin, and resveratrol derivatives to understand binding mode assessment: a small-molecule checkmate to cancer therapy. J Mol Model 26(9):242–54. doi: 10.1007/s00894-020-04488-0 10.1007/s00894-020-04488-0 32816149

[22] Lokhande KB, Nagar S, Swamy KV (2019). Molecular interaction studies of Deguelin and its derivatives with cyclin D1 and cyclin E in cancer cell signaling pathway: the computational approach. Sci Rep.

[23] Yi F, Li L, Xu L, Meng H, Dong Y, Liu H, Xiao P (2018). In silico approach in reveal traditional medicine plants pharmacological material basis. Chin Med.

[24] Yang H, Lou C, Sun L, Li J, Cai Y, Wang Z, Li W, Liu G, Tang Y (2018) AdmetSAR 2. 0: web-service for prediction and optimization of chemical ADMET properties. Bioinformatics 1–2. 10.1093/bioinformatics/bty707/5085368 10.1093/bioinformatics/bty707 30165565

[25] Gao Y, Gao Y, Yan L, Huang Y, Liu F, Zhao Y, Cao L, Wang T, Sun Q, Ming Z, et al (2020) Structure of the RNA-dependent RNA polymerase from COVID-19 virus. Science (80- ) [Internet] 1–9. Available from: www.sciencemag.org 10.1126/science.abb7498 PMC7164392 32277040

[26] Pettersen EF, Goddard TD, Huang CC, Couch GS, Greenblatt DM, Meng EC, Ferrin TE (2004). UCSF chimera - a visualization system for exploratory research and analysis. J Comput Chem.

[27] Umar HI, Awonyemi IO, Abegunde SM, Igbe FO, Siraj B (2020) In silico molecular docking of bioactive molecules isolated from Raphia taedigera seed oil as potential anti-cancer agents targeting vascular endothelial growth factor receptor-2. Chem Africa:1–16 10.1007/s42250-020-00206-8

[28] Trott O, Olson AJ (2010) AutoDock Vina: improving the speed and accuracy of docking with a new scoring function, efficient optimization and multithreading. J Comput Chem 31(2):455–461. https://doi.org/10.1002/jcc.21334.AutoDock 10.1002/jcc.21334 PMC3041641 19499576

[29] Jin Z, Du X, Xu Y, Deng Y, Liu M, Zhao Y (2020). Structure of M pro from SARS-CoV-2 and discovery of its inhibitors. Nature.

[30] Zhao Y, Hu J, Song J, Zhao X, Shi Y, Jiang Y (2020). Exploration on Shufeng Jiedu capsule for treatment of COVID-19 based on network pharmacology and molecular docking. Chin Med.

[31] Cheng F, Li W, Zhou Y, Jie S, Wu Z, Liu G, Lee PW, Tang Y (2012). AdmetSAR: a comprehensive source and free tool for assessment of chemical ADMET properties. J Chem Inf Model.

[32] Daina A, Michielin O, Zoete V (2017). SwissADME: a free web tool to evaluate pharmacokinetics, drug-likeness and medicinal chemistry friendliness of small molecules. Sci Rep.

[33] Mitra K, Ghanta P, Acharya S, Chakrapani G (2020) Dual inhibitors of SARS-CoV-2 proteases: pharmacophore and molecular dynamics based drug repositioning and phytochemical leads. J Biomol Struct Dyn 1–14. 10.1080/07391102.2020.1796802 10.1080/07391102.2020.1796802 PMC7441782 32698693

[34] Khan A, Khan M, Saleem S, Babar Z, Ali A, Aziz A, Zain K (2020) Phylogenetic analysis and structural perspectives of RNA - dependent RNA - polymerase inhibition from SARs - CoV - 2 with natural products. Interdiscip Sci Comput Life Sci 10.1007/s12539-020-00381-9 10.1007/s12539-020-00381-9 PMC7332347 32617855

[35] Mohapatra S, Prasad A, Haque F, Ray S, De B, Ray SS (2015). In silico investigation of black tea components on α -amylase , α -glucosidase and lipase. J Appl Pharm Sci.

[36] Salentin S, Schreiber S, Haupt VJ, Adasme MF, Schroeder M (2015) PLIP: fully automated protein – ligand interaction profiler. Nucleic Acids Res Adv 1–5. doi: 10.1093/nar/gkv315 10.1093/nar/gkv315 PMC4489249 25873628

